# Retina‐Inspired X‐Ray Optoelectronic Synapse Using Amorphous Ga_2_O_3_ Thin Film

**DOI:** 10.1002/advs.202410761

**Published:** 2024-11-14

**Authors:** Huili Liang, Xiaoyan Tang, Hang Shao, Rui Zhu, Shizhi Deng, Xiaozhi Zhan, Tao Zhu, Jiwei Wang, Jihua Zhang, Guangyu Zhang, Zengxia Mei

**Affiliations:** ^1^ Songshan Lake Materials Laboratory Dongguan Guangdong 523808 China; ^2^ Institute of Physics Chinese Academy of Sciences Beijing 100190 China; ^3^ College of Physics Liaoning University Shenyang 110036 China; ^4^ School of Electronic Science and Engineering State Key Laboratory of Electronic Thin Films and Integrated Devices University of Electronic Science and Technology of China Chengdu 610054 China; ^5^ China Spallation Neutron Source Dongguan Guangdong 523803 China

**Keywords:** amorphous Ga_2_O_3_, oxygen vacancy, X‐ray imaging, X‐ray optoelectronic synapse

## Abstract

Machine vision techniques are widely applied for object identification in daily life and industrial production, where images are captured and processed by sensors, memories, and processing units sequentially. Neuromorphic optoelectronic synapses, as a preferable option to promote the efficiency of image recognition, are hotly pursued in non‐ionizing radiation range, but rarely in ionizing radiation including X‐rays. Here, the study proposes an X‐ray optoelectronic synapse using amorphous Ga_2_O_3_ (a‐Ga_2_O_3_) thin film. Boosted by the interfacial V_O_^2+^ defects and its slow neutralization rate, the enhanced electron tunneling process at metal/a‐Ga_2_O_3_ interface produces remarkable X‐ray‐induced post‐synaptic current, contributing to a sensitivity of 20.5, 64.3, 164.1 µC mGy^−1^ cm^−2^ for the 1st, 5th, and 10th excitation periods, respectively. Further, a 64 × 64 imaging sensor is constructed on a commercial amorphous Si (a‐Si) thin film transistor (TFT) array. The image contrast can be apparently improved under a series of X‐ray pulses due to an outstanding long‐term plasticity of the single pixel, which is beneficial to the subsequent image recognition and classification based on artificial neural network. The merits of large‐scale production ability and good compatibility with modern microelectronic techniques belonging to amorphous oxide semiconductors may promote the development of neuro‐inspired X‐ray imagers and corresponding machine vision systems.

## Graphical abstract

This article presents a retina‐inspired X‐ray optoelectronic synapse. The slow neutralization rate of V_O_^2+^ states in a‐Ga_2_O_3_ is utilized to contribute to the non‐volatile post‐synaptic current. Real‐time X‐ray image sensing, memorizing, and contrast‐enhancing functions are achieved on a 64 × 64 sensor array, effectively promoting the recognition efficiency of following artificial neutral network simulations.

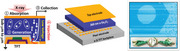

## 1 Introduction

X‐ray‐based machine vision systems are widely used in non‐destructive inspection for internal defects during modern industrial productions of large‐scale integrated circuits, components of precision instruments, Li‐ion batteries, packaged food, and so on.^[^ ^1^, ^2^ ^]^ During current continuous and real‐time image detection processes based on von Neumann architecture, considerable redundant data will be generated, posing challenges to the storage space, transmission speed, energy consumption, etc.^[^ ^3^, ^4^ ^]^ Retina in the human visual system can not only sense the light, but also memorize the information depending on the intensity and frequency of stimuli simultaneously. It possesses functions of image sensing, memorizing, and pre‐processing, and thus enables the visual cortex of the brain to process image information more efficiently. Various neuro‐inspired optical sensors have been reported working in non‐ionizing radiation range.^[^ ^5^, ^6^, ^7^, ^8^, ^9^, ^10^ ^]^ However, research on X‐ray neuromorphic sensors has received little attention so far.

It is well established that direct X‐ray detectors, which can convert absorbed X‐ray photons to electrical charges directly, are preferred for imaging applications because of high spatial resolution.^[^ ^11^ ^]^ To harvest enough electrical charges, all photodetectors, not just X‐ray detectors, need to absorb the incident photons, generate electron‐hole pairs (EHPs), and collect the mobile carriers as many as possible. Due to the high penetrability of X‐rays, much stricter requirements are imposed to the X‐ray detection materials, including heavy atoms, large thickness, low EHP creation energy, high resistance, and pure crystallinity together with strong radiation hardness.^[^ ^12^, ^13^ ^]^ Among the above multitudinous demands, a trade‐off between EHP creation energy and resistance as well as radiation hardness, caused by an opposite dependence on the semiconductor's bandgap,^[^ ^14^ ^]^ greatly limits further improvement of the device performance. Specifically, wide bandgap semiconductors are favorable regarding the enhanced radiation hardness and electrical resistance, but not the reduced EHP creation energy. Although many investigations have focused on them, the reported X‐ray‐induced photocurrents are usually in the level of several nano‐amperes,^[^ ^15^ ^]^ indicating significant room for improvement.

Despite of low X‐ray absorption efficiency and low carrier mobility, amorphous Se (a‐Se) is the most widely used direct X‐ray detection material considering its large‐area uniformity and relatively low cost.^[^ ^16^ ^]^ With the merits of higher thermal stability, much larger bandgap, and higher resistance as well as stronger radiation hardness, simple composition with no need for addition of expensive and rare In element, amorphous Ga_2_O_3_ (a‐Ga_2_O_3_) was proposed as an X‐ray sensitive candidate among various amorphous oxide semiconductors.^[^ ^17^, ^18^ ^]^ It should be noted that the X‐ray attenuation coefficient of Ga_2_O_3_ is a little bit lower than a‐Se, but higher than Si and comparable to other wide bandgap semiconductors, such as ZnO and GaN.^[^ ^19^, ^20^ ^]^ Importantly, a‐Ga_2_O_3_ can also be deposited uniformly on almost any large‐area substrates at room temperature,^[^ ^21^ ^]^ making it possible to fabricate X‐ray imagers directly on mature thin‐film‐transistor (TFT) technology, where TFTs serve as the switches to suppress crosstalk signals. Intriguingly, a nonlinear and nonvolatile current phenomenon was observed in a‐Ga_2_O_3_ detector under and after X‐ray irradiation, which was ascribed to ionized oxygen vacancy (V_O_) defects.^[^ ^18^ ^]^ Although surging interest in the role of V_O_ defects on a‐Ga_2_O_3_ ultraviolet (UV) optoelectronic synapse has been extensively reported,^[^ ^22^, ^23^, ^24^ ^]^ yet no attention has been paid to its influence on X‐ray range. Actually, similar phenomenon of X‐ray‐induced nonvolatile current has been reported in other oxide materials as well, TiO_2_, VO_2_, Sm_0.1_Bi_0.9_FeO_3_/Nb:SrTiO_3_ and Si/SrTiO_3_ hetero‐structures for instance.^[^ ^25^, ^26^, ^27^, ^28^ ^]^ This nonlinear and nonvolatile current provides a possibility to develop X‐ray optoelectronic synapses mimicking human retina as those working in UV or visible spectrum region.

In this work, X‐ray optoelectronic synapses have been constructed with a simple two‐terminal structure of Au/a‐Ga_2_O_3_/Au that exhibits typical synaptic behaviors such as light‐tunable and time‐dependent plasticity. The V_O_ defects at metal/semiconductor interface is of vital importance in the enhancement of electron tunneling process, which greatly relieves the contradiction between large bandgap and low EHP creation energy. Meanwhile, the de‐trapping process of the ionized V_O_ defects is very slow,^[^ ^22^ ^]^ allowing for the construction of synaptic X‐ray sensors. Using these X‐ray synapses, real‐time X‐ray image sensing, memorizing, and contrast enhancing functions have been demonstrated on a 64 × 64 amorphous Si (a‐Si) TFT matrix array. Further artificial neutral network simulations prove that time‐dependent image contrast enhancement can effectively improve the image quality and increase the processing efficiency of subsequent image recognition. This work opens a new view of large‐area neuromorphic X‐ray detectors with high image contrast, which may promote the image recognition efficiency for artificial intelligence applications.

## 2 Results and Discussion

### 2.1 Characterization of a‐Ga_2_O_3_ Thin Films

**Figure 1:**
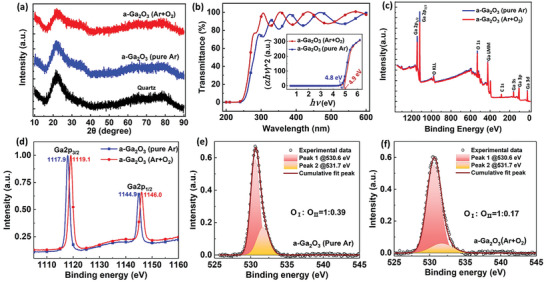
a) XRD curves of quartz substrate and a‐Ga_2_O_3_ thin films fabricated both in pure Ar and a mixture of Ar and O_2_. b) Transmittance spectra, c) XPS survey spectra, d) Ga 2p core level spectra, e,f) O 1s core level spectra of the above two films.

The a‐Ga_2_O_3_ thin films were deposited on quartz substrates in an atmosphere of pure Ar or mixture of Ar and O_2_ by radio frequency (rf) magnetron sputtering technique (more details can be found in Experimental Section). No characteristic peaks appear in X‐ray diffraction (XRD) curves, indicating the absence of a long‐range order (**Figure** **1**a) and amorphous structure nature. The transmittance spectra show a very small shrinkage of the bandgap energy for the film deposited in pure Ar (Figure 1b), which is probably originated from the expanded valence band tail states due to the existence of abundant V_O_ defects.^[^ ^18^ ^]^ Besides, the V_O_ defect concentration has been evaluated by X‐ray photoelectron spectroscopy (XPS) with the comparison of Ga 2p and O 1s core levels between a‐Ga_2_O_3_ thin films deposited in different atmospheres (Figure 1c–f). In the survey spectra of Figure 1c, only O, Ga and surface absorbed C elements exist. As exhibited in Figure 1d, the peak of Ga 2p_3/2_ shifts from 1117.9 to 1119.1 eV when oxygen flux is introduced into the rf‐sputtering chamber, indicating a more adequate oxidation state of gallium atoms in a‐Ga_2_O_3_. Meanwhile, the core level spectra of O 1s can be perfectly fitted by two sub‐peaks centered at 530.6 and 531.7 eV, which are named as O_I_ and O_II_ (Figure 1e,f). It is reported that O_I_ peak corresponds to the lattice oxygen of Ga_2_O_3_, and O_II_ peak is related with V_O_ defects. The addition of oxygen flux apparently leads to a decrease of the ratio between O_II_ and O_I_, demonstrating more V_O_ defects existing in the a‐Ga_2_O_3_ thin film deposited in pure Ar environment.^[^ ^22^ ^]^

**Figure 2:**
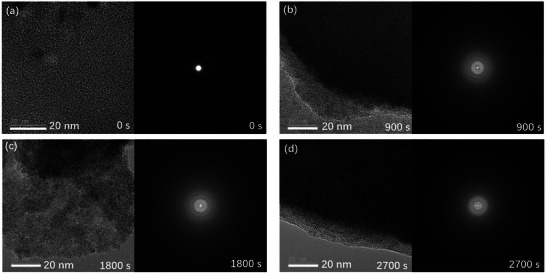
HRTEM images of a‐Ga_2_O_3_ (pure Ar) and the corresponding selected area electron diffraction (SAED) patterns after X‐ray irradiation for a) 0 s, b) 900 s, c) 1800 s, and d) 2700 s.

Then, the microstructure of a‐Ga_2_O_3_ thin film (pure Ar) before and after X‐ray irradiation has been characterized by high‐resolution transmission electron microscope (HRTEM) as shown in **Figure** **2**. There is no sign of structure change like poly‐crystallization after repeated radiation exposures as long as 2700 s, implying the admirable defect tolerance feature of amorphous solids.^[^ ^29^ ^]^ Inspiringly, lack of long‐range order in amorphous solids makes most atoms be considered as “defect atoms”, and no significant structure damage like point defects or dislocations will be aroused by repeated radiation exposures, implying a much stronger radiation hardness compared to its crystalline counterparts.^[^ ^30^, ^31^ ^]^ This feature lays the foundation for the development of long‐life high‐energy radiation detectors.

### 2.2 Synaptic X‐Ray Response of Single Device

**Figure 3:**
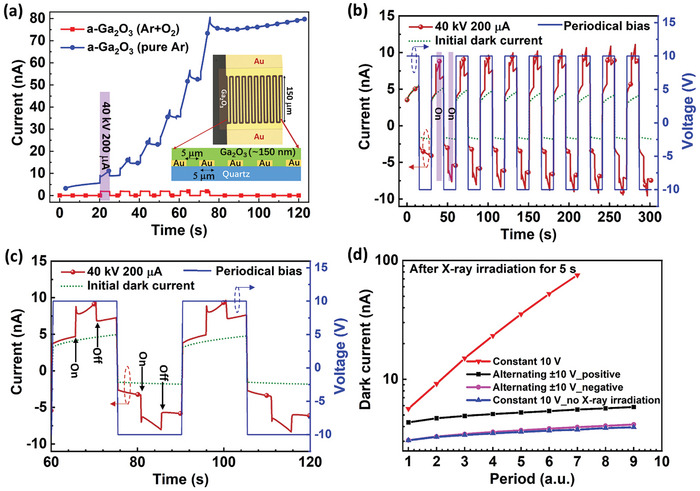
a) Time‐dependent X‐ray photoresponse under a constant 10 V bias. The insets illustrate the corresponding schematic device structure. b) Time‐dependent X‐ray photoresponse with ten periods under alternating ±10 V bias. This device was fabricated on a‐Ga_2_O_3_ thin film deposited in pure Ar. c) Two photoresponse periods of (b). d) Comparison of the dark current between different cases under constant bias and alternating bias.

Based on the above a‐Ga_2_O_3_ thin films, prototype X‐ray detectors with a coplanar metal‐semiconductor‐metal (MSM) structure were first constructed due to its much simpler fabrication process (**Figure** **3**). The insets of Figure 3a illustrate the schematic device structure. From the transient current curves under periodic X‐ray irradiations, it can be found that the device using a‐Ga_2_O_3_ thin film deposited in pure Ar displays a similar post‐synaptic response behavior in contrast to the sharp rise and down in the one deposited in a mixture of Ar and O_2_. The obvious discrepancy between these two devices is attributed to the V_O_ defects‐induced nonvolatile current.^[^ ^18^ ^]^ Significantly, the dark current can return back to the initial level immediately after applying an alternating ±10 V bias (Figure 3b,c), suggesting a successful electric suppression of the post‐synaptic current signals. Figure 3d compares the dark current variation after X‐ray irradiation under constant bias and alternating bias, clearly indicating the effectiveness of the alternating bias in suppressing the post‐synaptic current. An operational stability as long as 6300 s, i.e., 209 periods, can be found in Figure S1 (Supporting Information). ITO has also been adopted as the electrode (Figure S2, Supporting Information). Due to the different work function values between Au and ITO,^[^ ^32^, ^33^ ^]^ a higher dark current than Au/a‐Ga_2_O_3_ system appears apart from the similarly synaptic response performance. Therefore, Au electrode is preferred to ITO in view of its important role in lowering the detection limit.

**Figure 4:**
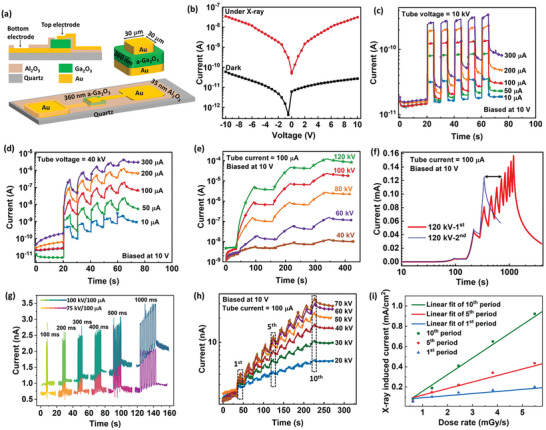
a) Schematic diagram of the vertical device structure. b) *I–V* curves of vertical a‐Ga_2_O_3_ X‐ray detector in dark and under X‐ray irradiation (40 kV/200 µA). c–h) Synaptic X‐ray response of the above vertical a‐Ga_2_O_3_ device under different X‐ray sources. The X‐ray source for (b), (c), and (d) is a miniature X‐ray tube from Moxtek, e,f) an open X‐ray tube from Comet, g,h) a micro‐focus X‐ray tube from Micro X‐ray Inc. i) Dependence of X‐ray induced current density on dose rate for different period irradiations as labeled in (h).

Further, a vertical MSM‐structured X‐ray detector was fabricated since it is more advantageous to high‐spatial resolution. **Figure** **4**a is the schematic diagram of the device structure. The initial *I–V* curves in dark and under X‐ray irradiation have been measured at first, where an apparent X‐ray photoresponse can be observed (Figure 4b). Time‐dependent X‐ray response was evaluated under different X‐ray sources. Detailed information about these X‐ray sources can be found in the Experimental Section. Figure 4c,d show the results excited by a compact miniature X‐ray tube with tube voltages of 10 kV and 40 kV, respectively. As the irradiation time increases, the X‐ray‐induced post‐synaptic current also increases, which is similar to the characteristic of bio‐synaptic behavior. In Figure 4e,f, an open X‐ray tube with higher tube voltages was adopted. The excitatory post‐synaptic current manifests more outstanding long‐term plasticity after X‐ray irradiation with higher and higher X‐ray intensity. The post‐synaptic current excited by 120‐kV X‐ray with three pulses in the second scan can reach to the same level as that of 6 pulses in the first scan (Figure 4f), quite resembling the re‐learning behavior of bio‐synapses. Moreover, X‐ray response has been demonstrated under short X‐ray pulses with period time ranging from 100 to 1000 ms (Figure 4g). Short‐term and long‐term synaptic plasticity can be modulated by the X‐ray dose rate and X‐ray pulse width synergistically. In addition, energy‐dependent long‐term synaptic plasticity was recorded by using monochromatic X‐ray photons from synchrotron radiation (Figure S3, Supporting Information). The device was irradiated by the monochromatic X‐ray photons of 20 keV and 10 keV (≈10^12^ photons/s) sequentially under a bias of 10 V. Energy‐dependent long‐term synaptic plasticity was recorded as shown in Figure S3a (Supporting Information). It should be noticed that the dark current can recover from a high level of ≈10^−7^ A to the initial ≈10^−9^ A after a fast *I–V* loop scan (Figure S3b, Supporting Information).

The X‐ray response sensitivity seems time‐dependent and has been estimated for a rough comparison with the values reported in the literatures. X‐ray‐induced current, defined as the difference of the currents when X‐ray source is off and on in the same irradiation period, has been extracted from the 1st, 5th, and 10th durations as labeled in Figure 4h. The corresponding dose rates were determined as illustrated in Note 1, Supporting Information. The linear fittings of the current curves against the X‐ray dose rate (Figure 4i) give a sensitivity value as 20.5, 64.3, 164.1 µC mGy^−1^ cm^−2^ for the 1st, 5th, and 10th periods, respectively. Clearly, the increased sensitivity originates from the time‐dependent X‐ray response, further illustrating the long‐term plasticity of the a‐Ga_2_O_3_ X‐ray optoelectronic synapse. A comparison has been made with recent reports^[^ ^11^, ^15^, ^16^, ^34^, ^35^, ^36^, ^37^, ^38^, ^39^, ^40^ ^]^ exhibiting a positive performance regarding sensitivity, thickness, and operation voltage (Figure S5, Supporting Information). As will be discussed in the following, electron tunneling process may be boosted at Au/a‐Ga_2_O_3_ interface, resulting in a decent X‐ray‐induced current and photoconductive gain despite of low X‐ray absorbance in such a thin a‐Ga_2_O_3_ film. Any comparison with typical photoconductive X‐ray detectors without gain may be unfair, but it still provides a benchmark to evaluate the response behavior of our synaptic X‐ray sensor.

### 2.3 Mechanism of X‐Ray‐Induced Post‐Synaptic Current

**Figure 5:**
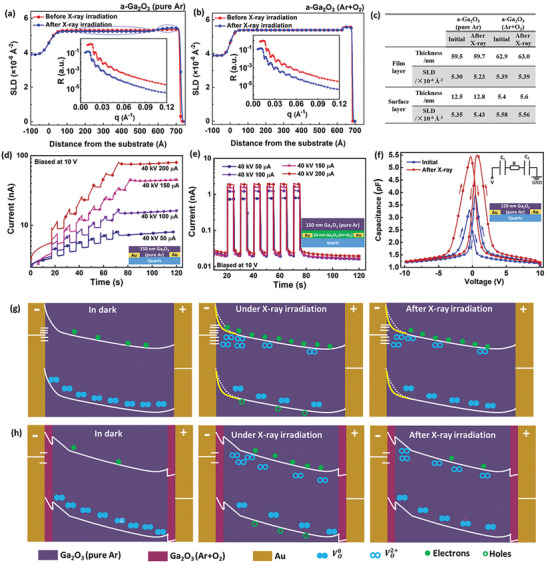
NR analysis of a‐Ga_2_O_3_ thin films before and after X‐ray irradiation. a,b) SLD as a function of the distance from the substrate toward the film surface for a‐Ga_2_O_3_ thin film deposited in pure Ar and in a mixture of Ar and O_2_, respectively. The insets are the experimental data and their corresponding fitting curves. c) The extracted parameters from NR fitting curves. d) Time‐dependent X‐ray photoresponse of the a‐Ga_2_O_3_ (pure Ar) device. e) Time‐dependent X‐ray photoresponse of the reference device with 20‐nm‐thick a‐Ga_2_O_3_ (Ar+O_2_) insertion layer. f) C–V measurements before and after X‐ray illumination probed with a sweeping bias of 100 mV at 10 kHz. g,h) Schematic band diagrams of Au/a‐Ga_2_O_3_ (pure Ar)/Au and Au/a‐Ga_2_O_3_ (Ar+O_2_)/a‐Ga_2_O_3_ (pure Ar)/a‐Ga_2_O_3_ (Ar+O_2_)/Au devices before, under, and after X‐ray irradiation, respectively.

The mechanism of X‐ray‐induced current was first disclosed by investigating the X‐ray irradiation on a‐Ga_2_O_3_ thin films using neutron reflection (NR) measurements. NR analysis, with the merit of detecting light elements including oxygen sensitively and nondestructively,^[^ ^41^ ^]^ was performed on a‐Ga_2_O_3_ thin films before and after X‐ray irradiation (**Figure** **5**a,b), respectively. The films were irradiated by the X‐ray tube of Moxtek with a distance of 5 mm for 60 min. The tube voltage is 40 kV and the tube current is 200 µA. A two‐layer model, including the main film layer and the surface layer, was used to fit the experimental curves. The fitting parameters are listed in the table shown in Figure 5c. The total fitted thickness is 72, 72.5, 68.3, and 68.6 nm, agreeing well with the designed thickness. Scattering length density (SLD), the other fitted parameter, is the product of the atom density (named n) and the coherent scattering length (called b) and can be considered as an indicator of the film composition.^[^ ^42^ ^]^ The SLD value of the film deposited in pure Ar is smaller than the one deposited in the mixture gas environment with O_2_, corroborating a lower V_O_ concentration in the latter. Besides, the SLD value is higher in the surface layer for both samples, which may be related with the surface absorbed oxygen,^[^ ^43^ ^]^ demonstrating the high sensitivity of NR technique. More importantly, after X‐ray irradiation, a clear decrease of the SLD value can be only found in the main film layer deposited in pure Ar, implying a possibility that more V_O_ defects have been created by X‐ray irradiation. Same X‐ray irradiation for 60 min was executed on the device without external electrical bias. After X‐ray irradiation, the dark current still stays at a higher level (Figure S6, Supporting Information) due to the high deionization barrier of V_O_^2+^ in a‐Ga_2_O_3_^[^ ^22^ ^]^. Moreover, the dark current cannot return to the initial level even after keeping the device in the dry cabinet for 50 days, further suggesting creation of new V_O_ defects under long‐term X‐ray irradiation.

A reference device was fabricated with a 20 nm a‐Ga_2_O_3_ (Ar+O_2_) insertion layer between Au and a‐Ga_2_O_3_ (pure Ar). Compared with the single‐layer device, the reference one demonstrates a lower photocurrent and quicker recovery speed after X‐ray source is off (Figure 5d,e). A similar response performance was observed in the vertical MSM structure with two 20 nm a‐Ga_2_O_3_ (Ar+O_2_) layers on the top and at the bottom of the main a‐Ga_2_O_3_ (pure Ar) layer, respectively (Figure S7, Supporting Information). Considering the significant difference of V_O_ defect density between the a‐Ga_2_O_3_ (pure Ar) and a‐Ga_2_O_3_ (Ar+O_2_) films, it can be discerned that V_O_ defect and Au/Ga2O_3_ interface synergistically contribute to the enhanced X‐ray response performance of the a‐Ga_2_O_3_ (pure Ar) thin‐film detector.

Ex situ C‐V characterization was carried out to explore the influence of X‐ray irradiation on defect and interface status thanks to the nonvolatile post‐synaptic current lasting for hundreds of seconds with a slow decay tendency (Figure S8, Supporting Information). Figure 5f displays the C‐V curves before and after X‐ray irradiation. The symmetric MSM structure with two back‐to‐back Schottky contacts can be simplified with a model of a resistor sandwiched by two capacitors as depicted in the inset. The total capacitance coming from the two tandem capacitors C_1_ and C_2_ will generate two symmetric peaks near zero bias.^[^ ^44^ ^]^ In our case, however, only one peak can be distinguished in both clockwise and anticlockwise voltage scans. These results can be reasonably interpreted with the accumulation of V_O_^2+^ at the negatively biased electrode,^[^ ^45^ ^]^ which breaks the original symmetry of the two identical contacts. As a result, the width of the depletion region will shrink compared with the initial one,^[^ ^46^ ^]^ leading to a higher capacitance as shown in Figure 5f. Importantly, the narrower depletion region increases the electrons’ tunneling probability through the interface barrier and gives rise to a larger current.^[^ ^47^ ^]^

Based on the above discussion, the carrier transport properties in a‐Ga_2_O_3_ X‐ray detectors are schematically illustrated in Figure 5g,h. Under X‐ray irradiation, neutral V_O_ defects in a‐Ga_2_O_3_ (pure Ar) will be ionized to V_O_^2+^ apart from the generation of electron‐hole pairs. Some holes will be trapped by V_O_ defects, also leading to the formation of V_O_^2+^. The interfacial V_O_^2+^ defects result in a narrower depletion width than the dark condition, as indicated by the yellow line in the middle image of Figure 5g. It increases the electrons’ tunneling probability from the external circuit, giving rise to a competent X‐ray response current. The bulk V_O_^2+^ defects promote the electrons’ hopping ability in a‐Ga_2_O_3_ layer as well. These two elements facilitate the reduction of bias voltage to a considerable degree. Due to the high deionization barrier of V_O_^2+^ states and the constraint of electric static potential, furthermore, the distribution of V_O_^2+^ defects is preserved and the dark current remains at a high level after switching off the X‐ray source (the right image of Figure 5g). For a better comparison, the initial depletion width of the Au‐Ga_2_O_3_ interface has been outlined by the dashed white lines in the last two images of Figure 5g. However, the 20 nm a‐Ga_2_O_3_ (Ar+O_2_) layer with much fewer V_O_ defects greatly prohibits the interface evolution and the occurrence of X‐ray‐induced nonvolatile photocurrent (Figure 5h). Taken overall, the utilization of electron tunneling process boosted by the V_O_^2+^ defects provides a strategy to resolve the contradiction between large bandgap and low EHP creation energy. Meanwhile, the slow de‐trapping process of V_O_^2+^ remembers the incident X‐ray photons, allowing for the construction of synaptic X‐ray sensors.

### 2.4 Imaging Performance of the X‐Ray Sensors

**Figure 6:**
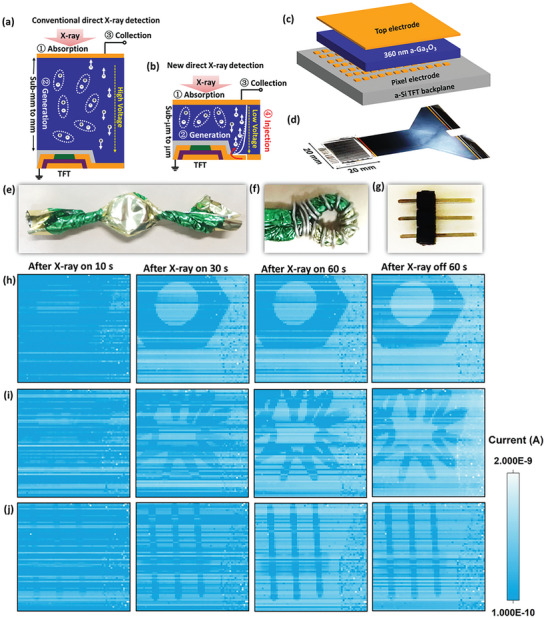
a) Conventional direct X‐ray detection process including absorption, generation, and collection. b) New direct X‐ray detection mode including an electron tunneling process. c) Schematic structure of the new X‐ray imaging sensor using a‐Ga_2_O_3_ thin film with a thickness of 360 nm. d) Photograph of the completed X‐ray imaging sensor. e–g) Photographs of a screw in a piece of candy paper, a tin wire wrapping around the candy paper, and an electronic pin. h–j) The imaging evolution of the above three objects irradiated for different times and their memorizing effect after X‐ray off for 60 s.

As mentioned previously, a‐Ga_2_O_3_ thin film can be deposited uniformly on almost any large‐area substrates at room temperature, giving us the chance to fabricate crosstalk‐free imaging sensor based on mature TFT technology. **Figure** **6**a displays the conventional direct X‐ray detection driven by the TFT, where absorption, generation, and collection processes are mainly responsible for the X‐ray‐induced current. In this case, quite thick photoconductive materials with hundreds of micrometers are usually welcome, elevating the difficulty in improving the device performance. Here, we propose a new strategy with the utilization of the boosted electron tunneling process by V_O_^2+^ defects (Figure 6b). In this new detection mode, the X‐ray‐induced current can be efficiently enhanced even in a thin film with large EHP creation energy. Figure 6c is the schematic structure of the X‐ray imaging sensor, where 360 nm Ga_2_O_3_ thin film was directly deposited on a commercial a‐Si TFT matrix by rf magnetron sputtering. TFT matrix defines the pixel size and suppresses the crosstalk signals. Figure 6d is the photograph of the completed device. A readout integrated circuit for TFT arrays was applied to record the current values using a gate voltage of ±10 V and a drain voltage of 4 V. The scan speed was 10.9 frames per second. The resolution of the a‐Ga_2_O_3_ array detector was tested at first by using an X‐ray line‐pair card with three lines at different spacing. As a direct‐conversion X‐ray detector, it can distinguish the line spacing up to 1.6 lp/mm (Figure S9, Supporting Information), implying a high spatial resolution. It should be noted that the resolution was restricted by the pixel size of the a‐Si TFT (200 × 200 µm^2^) in our case. Further, noise suppression effect was demonstrated by imaging different objects as shown in Figure 6e–g. The X‐ray source irradiated the device with a condition of 70 kV/150 µA during every “on” period, and the post‐synaptic current of each pixel was acquired for 60 s after X‐ray source off. The imaging pictures were drawn based on the raw current data (Figure 6h–j). The longer the array was irradiated, the better the image quality became, which is attributed to the outstanding long‐term synaptic plasticity of the single pixel. More remarkably, the imaging details were memorized and retained even for 60 s after switching off the X‐ray source, manifesting the potential for fulfilling both sensing and storage functions in the same one imaging sensor.

It may be noticed that the emergence of object image needs a few seconds herein, implying that the detector's sensitivity must be optimized further to facilitate more carriers’ charging in a much shorter time period. Increasing the thickness of a‐Ga_2_O_3_ thin film is one of the most effective routes as shown in Figure S10 (Supporting Information). Another key point is that an extra reset circuit is necessary to make the bio‐synaptic‐like X‐ray sensor quickly return to standby state and possibly deal with dynamic objects. On a positive note, however, the a‐Ga_2_O_3_ imaging detector presents neuromorphic preprocessing abilities which has been developed in non‐ionization radiation spectra but seldom in X‐ray range.

### 2.5 Simulations of Neuromorphic Image Recognition

**Figure 7:**
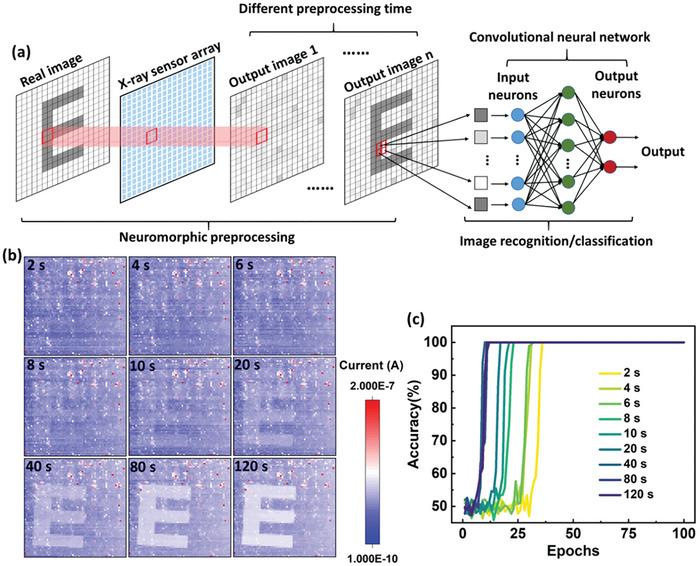
a) Schematic illustration of the X‐ray neuromorphic visual system consisting of the X‐ray sensor array for image capture and preprocessing as well as an artificial neural network for image recognition. b) Presentative images obtained by the 64 × 64 array detector under different irradiation time. c) Comparisons of the image recognition rate under different preprocessing time.

To explore the possible application of the X‐ray optoelectronic synapse in machine vision, a neuromorphic artificial visual system was established including the 64 × 64 sensor array and convolutional neural network (CNN). The former component is meant to capture and preprocess the image information as the common sensory neurons do in the retina of the human visual system, and the latter to execute the image recognition task like the visual cortex in human brain. **Figure** **7**a displays the frame work of the X‐ray artificial visual system, where LeNet architecture, a classic model, is chosen as the neural network.^[^ ^48^ ^]^ More details can be found in Figure S11 and Table S1 (Supporting Information).

A steel metal mask with a capital “E” was used for the target detection and identification measurements. A series of “E” images were recorded by the 64 × 64 sensor array. The image contrast is obviously enhanced while the irradiation time increases, where 9 images are selected as representatives (Figure 7b). Random Guassian noises with a mean of 9 and standard deviation of 5 were introduced into the grayscale images in Figure 7b, resulting in 900 new images for each one. These images together with the background of the array sensor construct an image database for the training and recognition processes of the neuromorphic artificial visual system. Detailed information of the database is given in Figure S12 (Supporting Information). Figure 7c illustrates the recognition rate of the visual system with different irradiation time. Obviously, an improved recognition efficiency can be achieved due to the higher image contrast as the irradiation time increases. Different running cycles have been performed as shown in Figure S13 (Supporting Information), indicating a good repeatability of the results. Additionally, a goal of classifying different objects’ images shown in Figure 6e–g has been successfully accomplished by using the same model (Figure S14, Supporting Information), where the recognition efficiency is also apparently boosted as the irradiation time increases.

## 3 Conclusion

To conclude, a new paradigm for direct X‐ray detection has been realized on a‐Ga_2_O_3_ thin film, where V_O_^2+^ defects and interface confinement are revealed playing a synergetic role in the collection of X‐ray‐induced nonvolatile current. X‐ray neuromorphic imaging sensor has been successfully realized on a commercial 64 × 64 TFT matrix. The image contrast is improved efficiently due to the outstanding long‐term plasticity of the single pixel, which further increases the subsequent image recognition and classification efficiency. This work opens a new vision of large‐area and low‐cost neuromorphic X‐ray imagers, which may promote the application of X‐ray‐based machine vision in the field of modern industry.

## 4 Experimental Section

### Film Growth and Device Fabrication

The a‐Ga_2_O_3_ thin films were deposited by radio frequency (rf) sputtering technique, where a Ga_2_O_3_ ceramic target with 4N purity was adopted. The sputtering power and pressure were 60 W and 0.4 Pa, respectively. During the sputtering process, the flux of Ar gas was kept at 10 sccm while O_2_ flux fixed at 0 and 1.0 sccm for synaptic and normal devices, respectively.

The X‐ray detectors were fabricated on quartz substrates with a metal‐semiconductor‐metal (MSM) structure. For the coplanar device, ITO or Au electrodes were deposited at room temperature first. The sputtering power and pressure were 50 W and 0.4 Pa, respectively. After UV lithography, the electrodes were chemically wet etched to form 50 pairs of interdigital electrodes with a width of 5 µm, an interval of 5 µm, and a length of 150 µm. Next, a‐Ga_2_O_3_ thin films were deposited on the patterned electrodes at room temperature via rf‐sputtering. Conventional lithography and etching process was carried out again to define the active area of a‐Ga_2_O_3_ thin films with 175 µm in width and 1020 µm in length.

For vertical MSM device, sandwiched Au/a‐Ga_2_O_3_/Au thin films were deposited sequentially at room temperature. Then, combined with UV lithography and chemical wet etching, the above three layers were etched from top to bottom to form a 30 × 30 µm active area. After an insulating Al_2_O_3_ thin film prepared by atomic layer deposition (ALD) on the surface, large metal pads were added for the probe contact during electrical measurements.

For the 64 × 64 array detector, the a‐Ga_2_O_3_ thin film was directly deposited on the commercial a‐Si TFT matrix by rf sputtering technique, where the uncovered drain electrode of the TFT was used as the bottom contact of a‐Ga_2_O_3_. Then, the top contact electrode was deposited on a‐Ga_2_O_3_ thin film. After finishing the film deposition process, a piece of flexible printed circuit was welded on the matrix for subsequent imaging tests.

### Material Characterization

The film thickness was measured by a step profiler (KLA‐Tencor P‐6 stylus profiler) after a selective chemical etching. The X‐ray diffraction patterns were recorded by the X‐ray diffractometer (Bruker, D8 Advance) with normal θ‐2θ scanning mode. The optical transmittance of a‐Ga_2_O_3_ thin films was measured using a UV−vis–NIR spectrometer (Hitachi spectrophotometer UH4150). X‐ray photoelectron spectroscopy (XPS, ESCALAB Xi+, ThermoFisher) was used to evaluate the chemical bonding states of the a‐Ga_2_O_3_ thin films prepared in pure Ar and a mixture of Ar and O_2_, respectively. To get the high‐resolution transmission electron microscopic (HRTEM) images, water‐soluble NaCl substrates were used for a‐Ga_2_O_3_ thin film deposition. After X‐ray irradiation for different times, NaCl substrates were dissolved in deionized water and the left a‐Ga_2_O_3_ thin films were transferred onto the copper grids for TEM (JEOL, JEM‐F200) observations. Neutron reflection (NR) spectra were recorded using the non‐polarized mode of multipurpose reflectometer, a time‐of‐flight neutron reflectometer (2.5 < λ < 7 Å), at China Spallation Neutron Source (CSNS).

### Device Characterization

Room‐temperature electrical and optoelectronic measurements for the single device were performed in air using a Keithley 6487 or Keithley 2636B source meters. Different X‐ray sources have been used to irradiate the single device, including a compact X‐ray tube with a W anode (Moxtek), two micro‐focus X‐ray tubes (one is from the company of Micro X‐ray Inc. with the model of Microbox 100, the other is from the company of Comet X‐ray with the model of FXE 160 micro) as well as monochromatic X‐ray beam from X‐ray imaging station (4W1A) in Bejing Synchrotron Radiation Facility (BSRF). C‐V measurements were carried out using a Keysight B1500A semiconductor parameter analyzer equipped with a Lake Shore probe station. The imaging data for the 64 × 64 array were collected by a readout integrated circuit under irradiation of the micro‐focus X‐ray source (Microbox 100, Micro X‐ray Inc.).

## Conflict of Interest

The authors declare no conflict of interest.

## Supporting information



Supporting Information

## Data Availability

The data that support the findings of this study are available from the corresponding author upon reasonable request.
