# Supplementary material for: Retina‐Inspired X‐Ray Optoelectronic Synapse Using Amorphous Ga2O3 Thin Film
Source: Adv Sci (Weinh). 2024 Nov 14;11(48):2410761. doi: 10.1002/advs.202410761 (PMC11906217; doi:10.1002/advs.202410761)
Supplement: Supplementary file 1 — Supporting Information [file ADVS-11-2410761-s001.docx]

Supporting Information

Retina-inspired X-ray optoelectronic synapse using amorphous Ga_2_O_3_ thin film

*Huili Liang*^*^*, Xiaoyan Tang, Hang Shao, Rui Zhu, Shizhi Deng, Xiaozhi Zhan, Tao Zhu, Jiwei Wang, Jihua Zhang, Guangyu Zhang, Zengxia Mei*^*^

Dr. H. L. Liang, X. Y. Tang, H. Shao, Dr. R. Zhu, S. Z. Deng, Prof. T. Zhu, Prof. G. Y. Zhang, Prof. Z. X. Mei

Songshan Lake Materials Laboratory, Dongguan, Guangdong 523808, China

Dr. H. L. Liang, Dr. R. Zhu, Prof. T. Zhu, Prof. G. Y. Zhang, Prof. Z. X. Mei

Institute of Physics, Chinese Academy of Sciences, Beijing 100190, China

X. Y. Tang, Prof. J. W. Wang

College of Physics, Liaoning University, Shenyang 110036, China

H. Shao, Prof. J. H. Zhang

School of Electronic Science and Engineering, State Key Laboratory of Electronic Thin Films and Integrated Devices, University of Electronic Science and Technology of China, Chengdu 610054, China

Dr. X. Z. Zhan, Prof. T. Zhu

China Spallation Neutron Source, Dongguan, Guangdong 523803, China

^*^E-mails: hlliang@iphy.ac.cn, zxmei@iphy.ac.cn


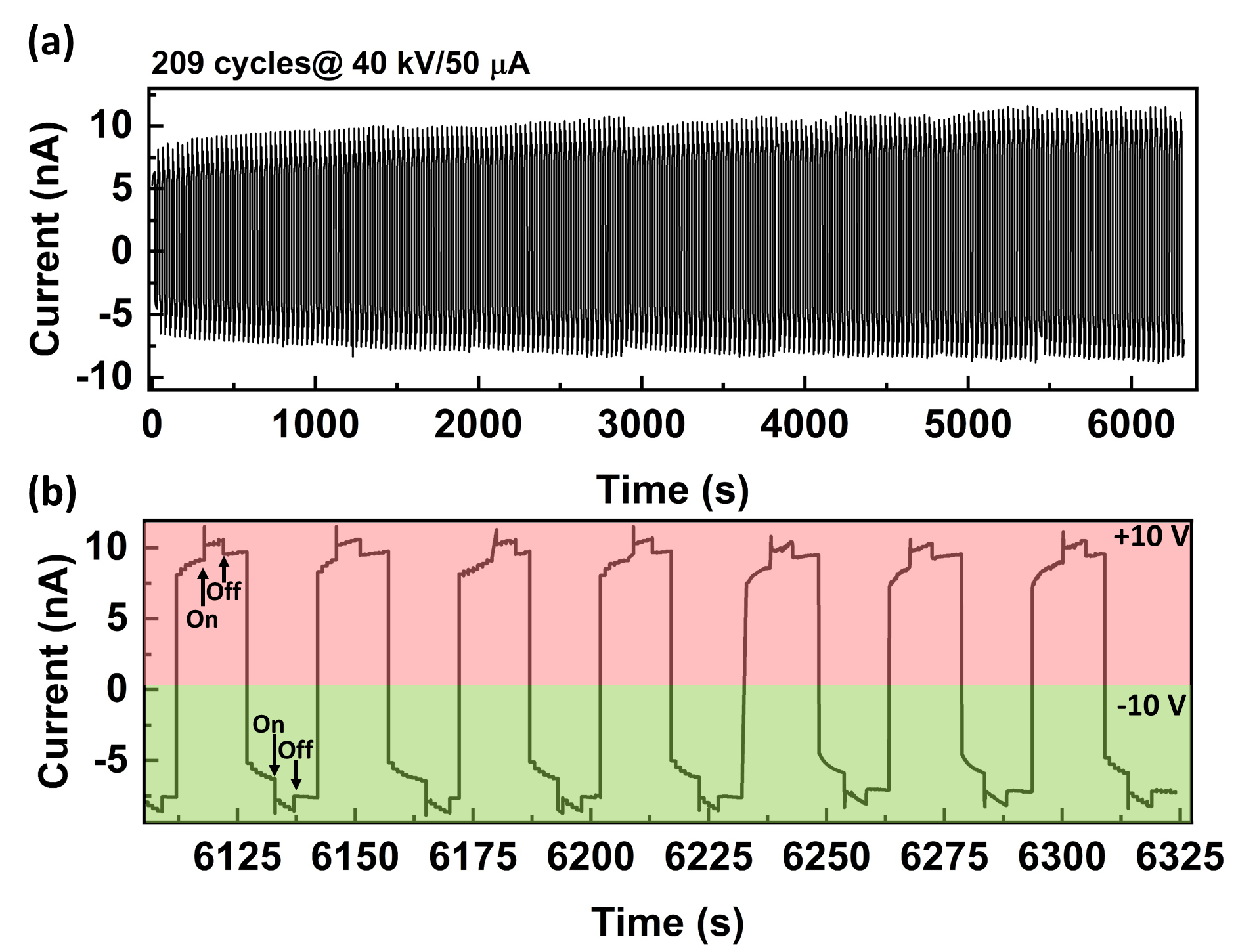


**Figure S1.** Operational stability over 209 periods under alternating ±10 V bias. (a) Time-dependent X-ray photoresponse with X-ray intensity of 40 kV/50 A. (b) The last 7 periods are demonstrated for clarity.


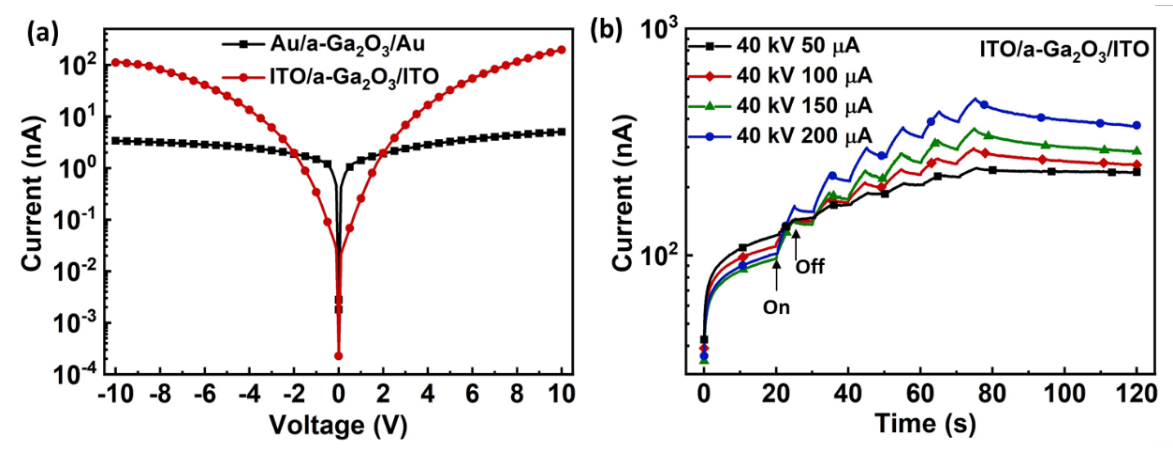


**Figure S2.** X-ray detection performance of a-Ga_2_O_3_ using buried ITO electrodes. (a) Comparison of the I-V curves in dark for the devices using Au and ITO electrodes. The deposition condition for a-Ga_2_O_3_ thin films and device dimension are the same in the two devices. (b) Time-dependent X-ray photoresponse of ITO/a-Ga_2_O_3_/ITO under a constant 10 V bias.


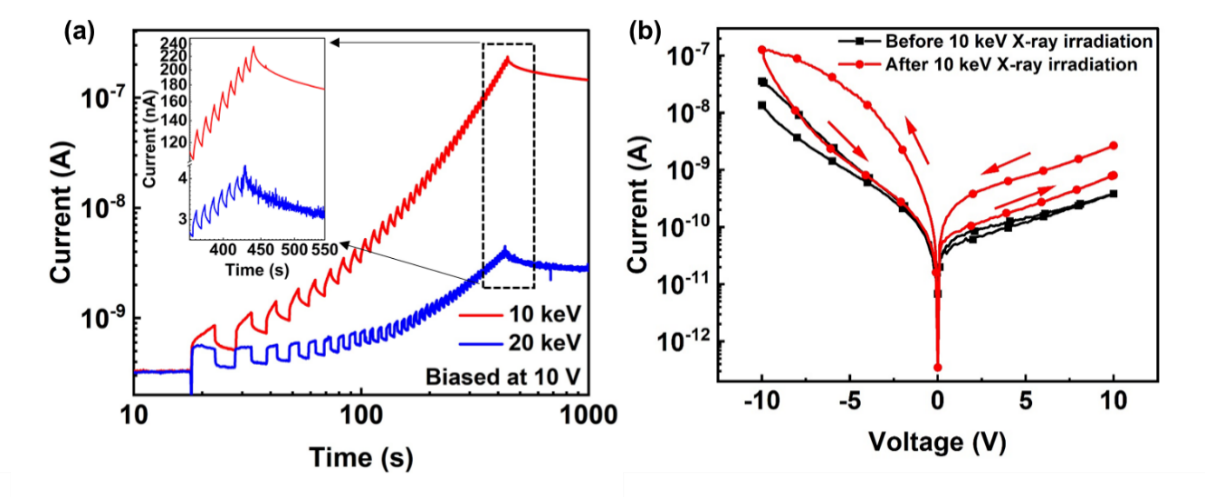


**Figure S3.** (a) Synaptic response of vertical MSM-structured a-Ga_2_O_3_ device using monochromatic X-ray sources from synchrotron radiation. (b) I-V curves in dark before and after 10 keV X-ray irradiation.

The dose rate from synchrotron radiation can be reasonably estimated based on its definition as shown in the following equation,

where Φ is the photon flux density, *A* is the active detecting area,* μ_m_* is the mass attenuation coefficient in air, *ρ* is the air density, *d* is the distance X-ray photons going through, and *ε_ph_* is the energy of each X-ray photon.

$D=\frac{\mathrm{dE}}{\mathrm{dm}}$ $=\frac{\Phi\times A\times[1-\exp(-\mu_{m}\times\rho\times d)] \times\varepsilon_{ph}}{\rho\times d\times A}$ (1)

Considering the fact that X-rays are highly penetrating, the above equation can be simplified as

$D=\frac{\mathrm{dE}}{\mathrm{dm}}$ $=\Phi\times\mu_{m}\times\varepsilon_{ph}$ (2)

Then, we can calculate the dose rate just after obtaining Φ, *μ_m_*, *ε_ph_*. The value of *μ_m_* is referred as about 10 cm^2^/g from the website of National Institute of Standards and Technology (https://physics.nist.gov/PhysRefData/XrayMassCoef/ComTab/air.html). Φ is roughly estimated as 6×10^9^ photons/s/mm^2^ according to the information provided by 4W1A-X-ray Imaging station in Beijing Synchrotron Radiation Facility (BSRF). Thus, the dose rate will be 9.5 Gy/s and 19.0 Gy/s for monochromatic X-ray photons with energy of 10 keV and 20 keV, respectively. As a result, the incident total dose during the measurents shown in Figure S3 is about 5.7 kGy.

**Note 1 Evaluation of X-ray dose rate at the position of the sample holder.**

The dose rate of the X-ray source (Microbox 100, Micro X-ray Inc.) was monitored by an ionization dosimeter (Radcal). Since the sensitive detection range of the dosimeter is above 40 kV, the tube voltage is set as 40, 50, 60, 70 kV. The tube current is set constantly at 100 A. The distance between the sample holder and the X-ray source is 6 cm. A 4 mm Cu plate is equipped behind the X-ray tube in order to attenuate the intensity of the X-ray beam. The dose rate detected by the dosimeter is 0.687, 2.385, 5.333 and 9.968 Gy/s for 40, 50, 60 and 70 kV, respectively.

To get the real dose rates of the naked X-ray tube, some calculation processes were carried out as illustrated below.

At first, the transmittance of X-ray photons after the 4 mm Cu plate was obtained by the equation of $T=\frac{I_{Cu}}{I_{0}}=exp\left[ -\left( \frac{\mu}{\rho} \right).\rho.t \right]$, where T is the transmittance, *I_Cu_* the X-ray intensity after Cu attenuation, *I_0_* the initial X-ray intensity, $\rho$ the mass density of Cu, t the thickness, and $\mu/\rho$ the mass attenuation coefficient given on the website of National Institute of Standards and Technology (NIST) as shown in Figure S4a (https://physics.nist.gov/PhysRefData/XrayMassCoef/ElemTab/z29.html).


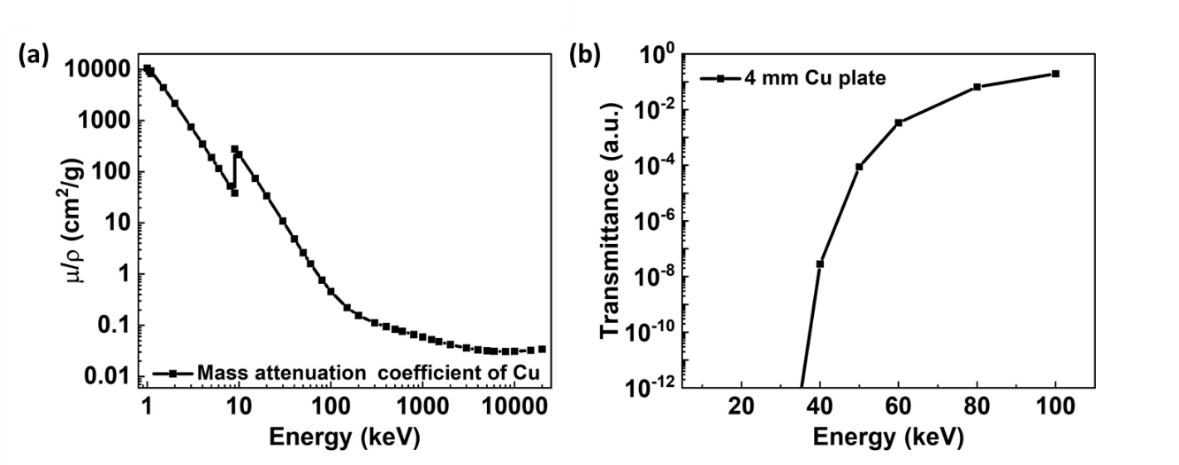


**Figure S4.** (a) Mass attenuation coefficient of Cu for X-ray photons with different energies. (b) Transmittance spectra of the X-ray photons after 4 mm Cu plate.

Inconsistent with the calculated transmittance as displayed in Figure S4b, the measured dose rates under a tube voltage of 40, 50, 60 and 70 keV, respectively, are in the same magnitude. Some leakage must happen because the illumination cone of the X-ray source is so large (95°) that the Cu plate (r = 12.5 mm) cannot block all of the X-ray photons. Therefore, the measured dose rate for the tube voltage of 70 kV is chosen to estimate the irradiation intensity from the naked tube.

Since the X-ray emission from the naked X-ray tube is non-monochromatic, the relationship of the irradiation intensity between the naked tube and the one filtered by the Cu plate should be defined as $\frac{I_{Cu}}{I_{0}}=\frac{\int_{20}^{70} \Phi(E)\alpha(E)dE}{\int_{20}^{70} \Phi(E)dE}$, where $I_{Cu}$ is the attenuated intensity, $I_{0}$ the primary intensity, $\Phi(E)$ the X-ray spectra of W tube accelerated by 70 kV with no filters and can be referred to the reported literature^1^, and$\alpha\left( E \right)$ the attenuation spectra of the X-ray photons by 4 mm Cu plate as shown in Figure S4a. Based on the above equation, only 1.333‰ X-rays can penetrate through such a thick Cu plate. Taking the measured dose rate of 9.968 Gy/s for 70 kV X-ray tube as $I_{Cu}$, $I_{0}$ will be ~7.48 mGy/s. It should be noted that the real intensity of the naked X-ray tube is lower than 7.48 mGy/s due to the leakage mentioned above. Nevertheless, this value can still be served as an evaluation base of the X-ray source used in this work.

Further, by using the empirical relationship of $I=k\times i\times U^{n}$, where *I* is the average Bremsstrahlung intensity, *k* the coefficient of proportionality as a function of the atomic number of the anode material and the power circuit of X-ray tube, *n* an empirical value usually taken as 2, *U* the applied tube voltage, and *i* the tube current^2^, we can get the dose rates at other tube voltages with the constant tube current of 100 A. Specifically, the dose rates for 20, 30, 40, 50, 60 kV are estimated to be 0.61, 1.37, 2.44, 3.81, 5.49 mGy/s, respectively.


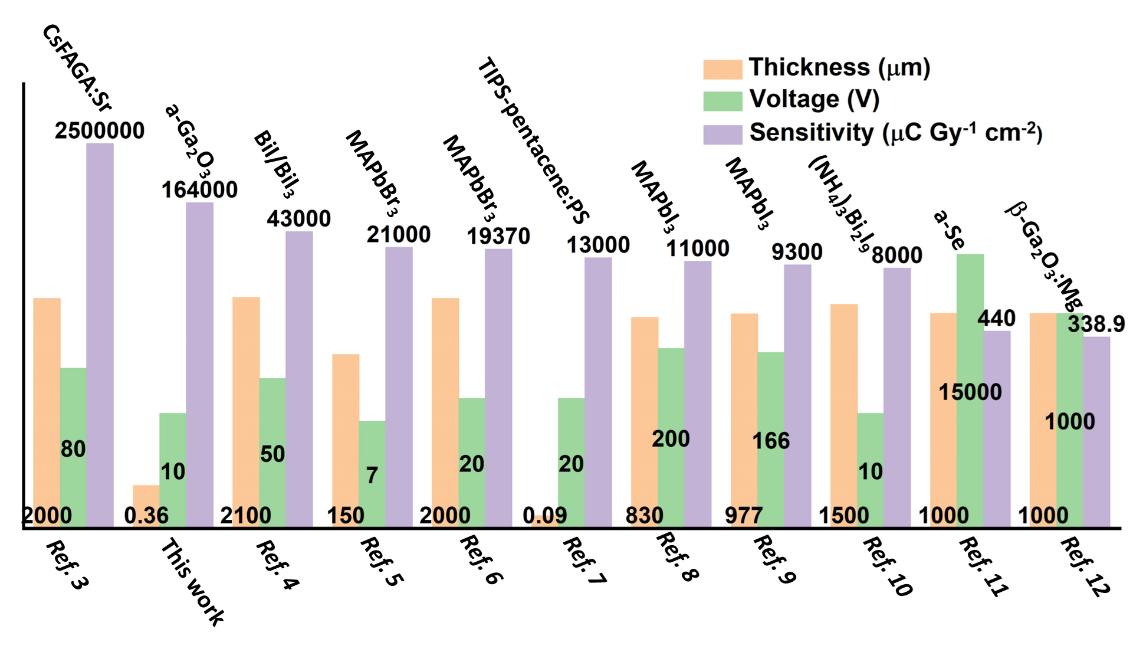


**Figure S5.** Comparison of sensitivity vs thickness & bias voltage between our results and the values reported in recent literatures^3-12^.


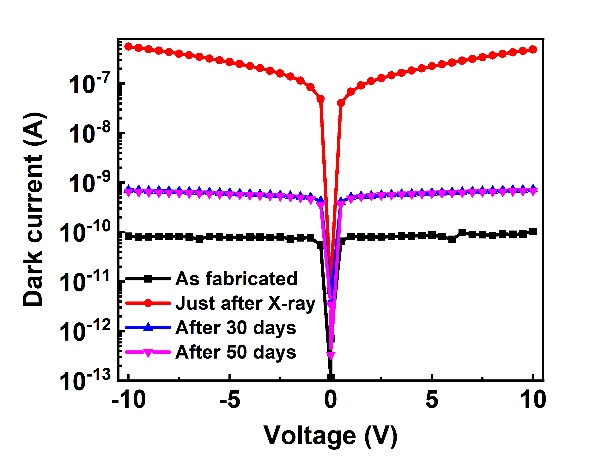


**Figure S6.** The dark current of a-Ga_2_O_3_ (pure Ar) device before and after X-ray irradiation (40 kV/200 A). The device was irradiated without external electrical bias by the X-ray tube of Moxtek with a distance of 5 mm for 60 min.


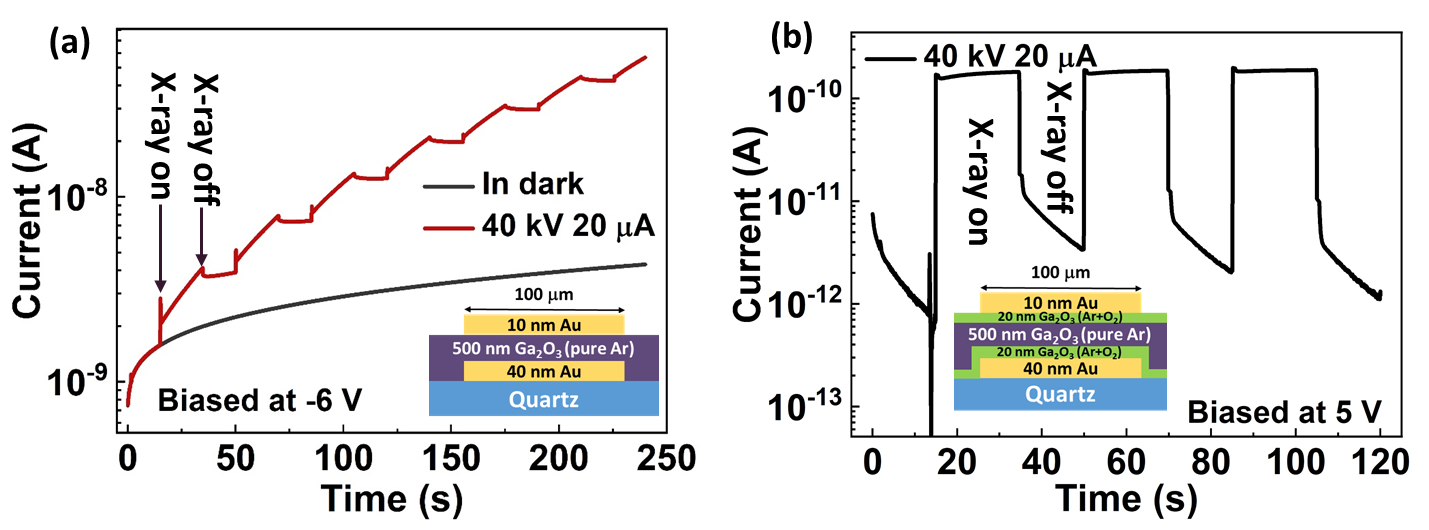


**Figure S7.** Temporal current curves under periodic X-ray irradiations on (a) vertical MSM-structured Au/a-Ga_2_O_3_ (pure Ar)/Au device and (b) vertical MSM-structured Au/a-Ga_2_O_3_/Au device with two 20 nm a-Ga_2_O_3_ (Ar+O_2_) layers on the top and at the bottom of the main a-Ga_2_O_3_ (pure Ar) layer, respectively. The inset shows the schematic device structure.


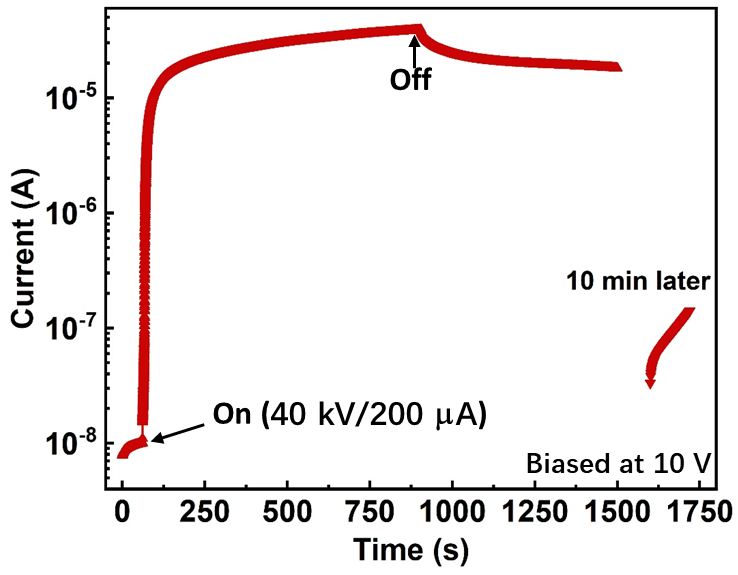


**Figure S8.** Nonvolatile X-ray-induced current of the a-Ga_2_O_3_ (pure Ar) device after X-ray illumination for 840 s.


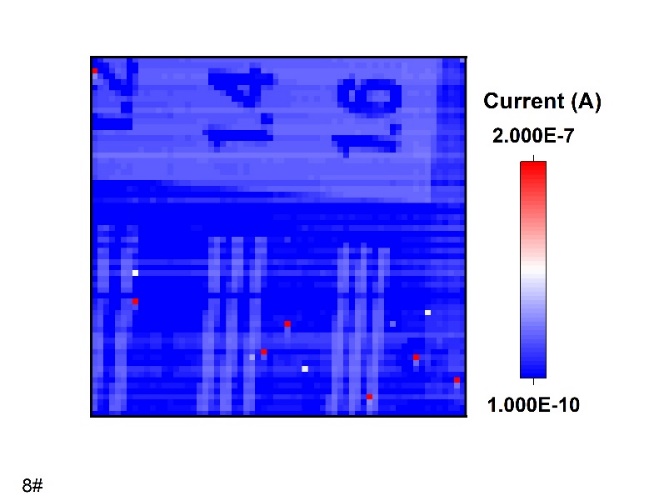


**Figure S9.** Spatial resolution of the X-ray image up to 1.6 lp/mm.


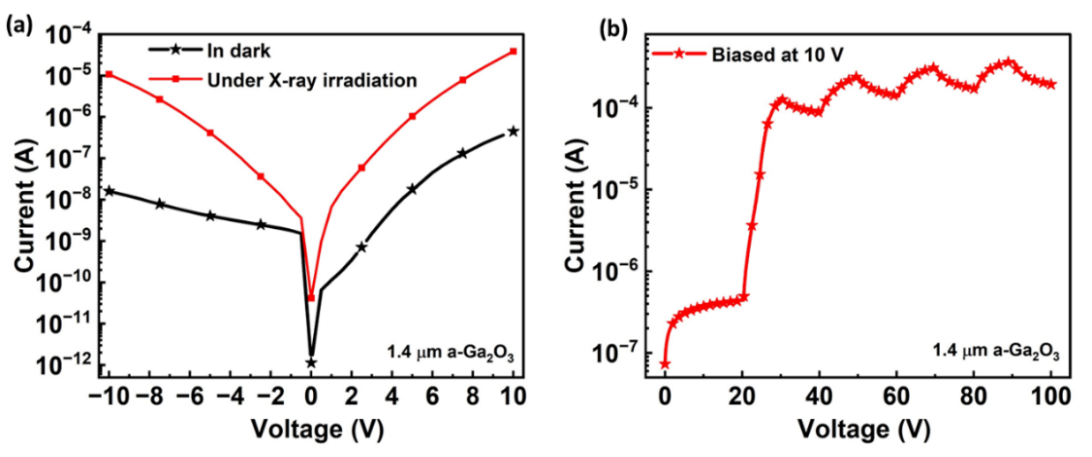


**Figure S10.** (a) I-V curves in dark and under X-ray irradiation of the vertical MSM-structured a-Ga_2_O_3_ X-ray detector. (b) The corresponding time-dependent X-ray photoresponse under a constant 10 V bias. The thickness of a-Ga_2_O_3_ is 1.4 μm.


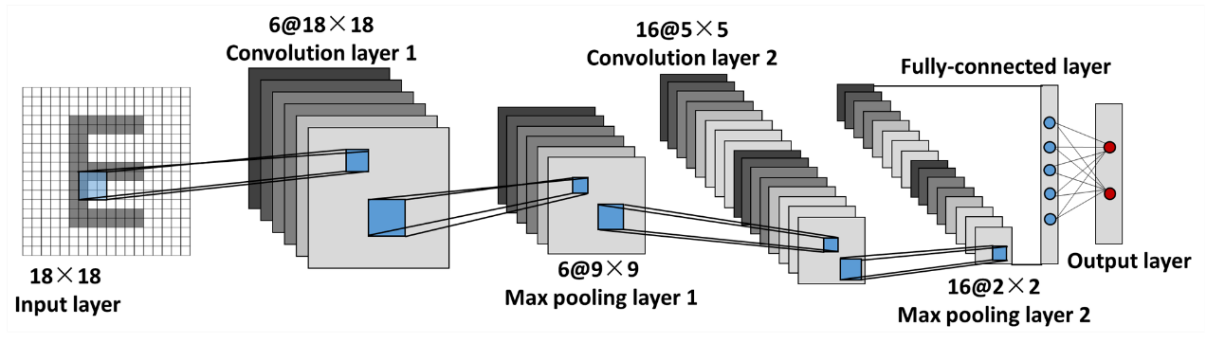


**Figure S11.** Illustration of convolutional neural network. The input is an image recorded by the 64×64 image sensor, the output is a probability over 2 possible outcomes.


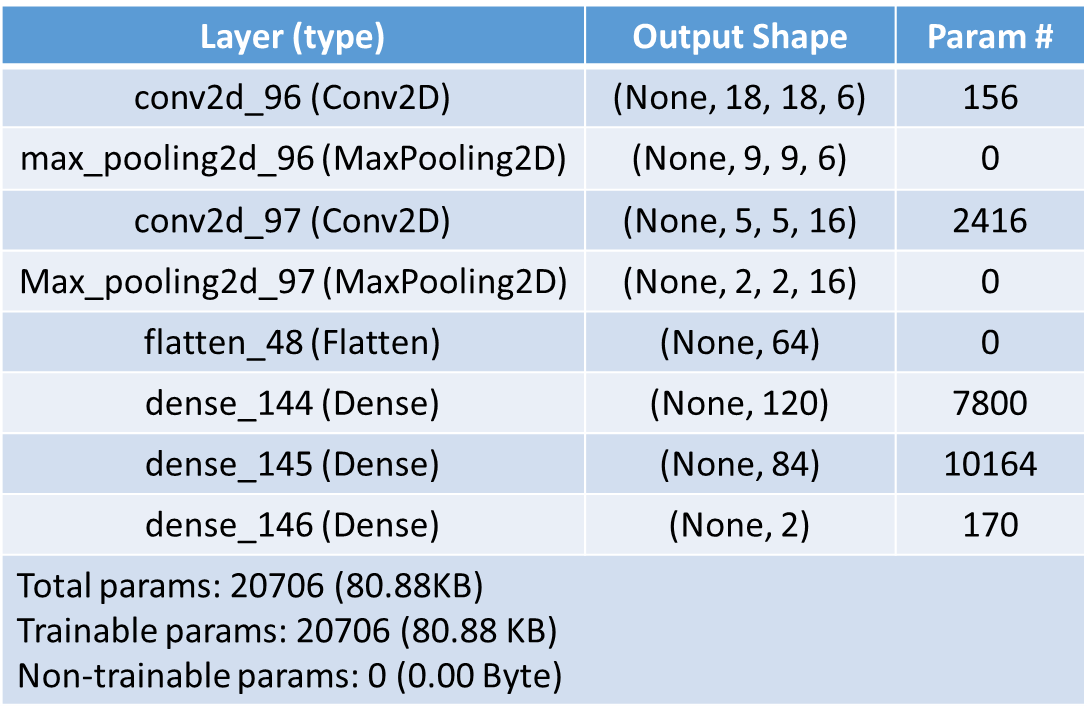


**Table S1.** Parameters involved during the training and testing processes.

The LeNet-based CNN structure comprises 7 layers, including two convolutional layers, two max pooling layers and three fully-connected layers as illustrated in Figure S11. Parameters involved during the training and testing processes are listed in Table S1. The input is an 18×18 grayscale image. The first convolutional layer has 6 kernels with a size of 18×18. The size of kernel is 5×5. Padding is added with a stride of (1, 1) during the convolutional process. This layer contains 156 trainable parameters. The first max pooling layer has 6 kernels with a size of 9×9. The size of pooling window is 2×2 and the stride is (2, 2). The second convolutional layer has 16 kernels with a size of 5×5. The size of kernel is 5×5. Padding is added with a stride of (2, 2) during the convolutional process. This layer contains 2416 trainable parameters. The second max pooling layer has 16 kernels with a size of 2×2. The size of pooling window is 2×2 and the stride is (2, 2). A flatten layer is inserted between the second max pooling layer and the fully-connected layers in order to transfer the two-dimensional data array into one-dimensional. The dimensional of next 3 fully-connected layers is 120, 84 and 2, respectively. Back-propagation (BP) algorithm is used for training and recognition processes. The activation function in convolutional layers is Sigmoid. The learning rate is set as a fix value of 0.003.

The image dataset in the training and recognition processes are generated in the following steps. At first, two pictures, one with the letter E captured by the array sensor and the other is the background noise of the array sensor, are converted into grayscale images with the gray values distributed between 0-255. Then, both of the two images are cropped into a small size of 18×18 by using the Lanczos filter algorithm with Python's Pillow library. At last, random Gaussian noises with a mean of 9 and standard deviation of 5 are introduced into the above two pictures. 900 new pictures are derived based on the three steps for the imager background and letter E, respectively. Figure S12 demonstrates some examples in the image database.

80% and 20% of the 1800 images are randomly selected as the training and testing set, respectively. The random selection rule is made by using the model of sklearn.model_selection in Python machine learning repository. The seed of the random number is set as 42.


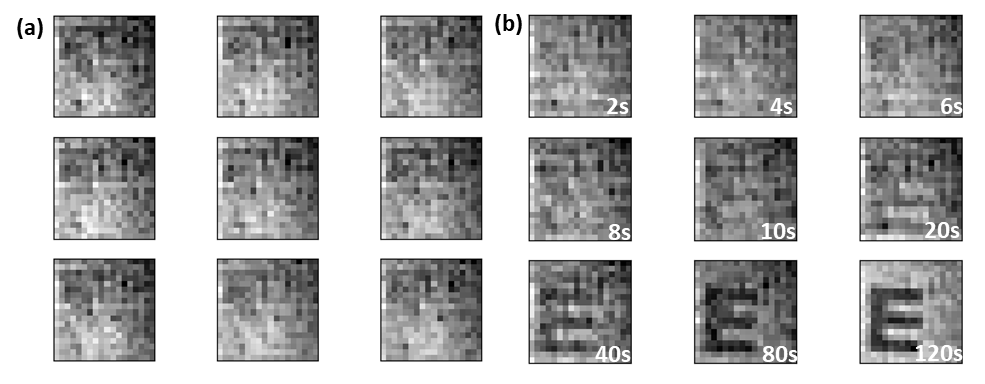


**Figure S12.** Demonstrations of examples in image dataset for imager background (a) and letter E (b).

**
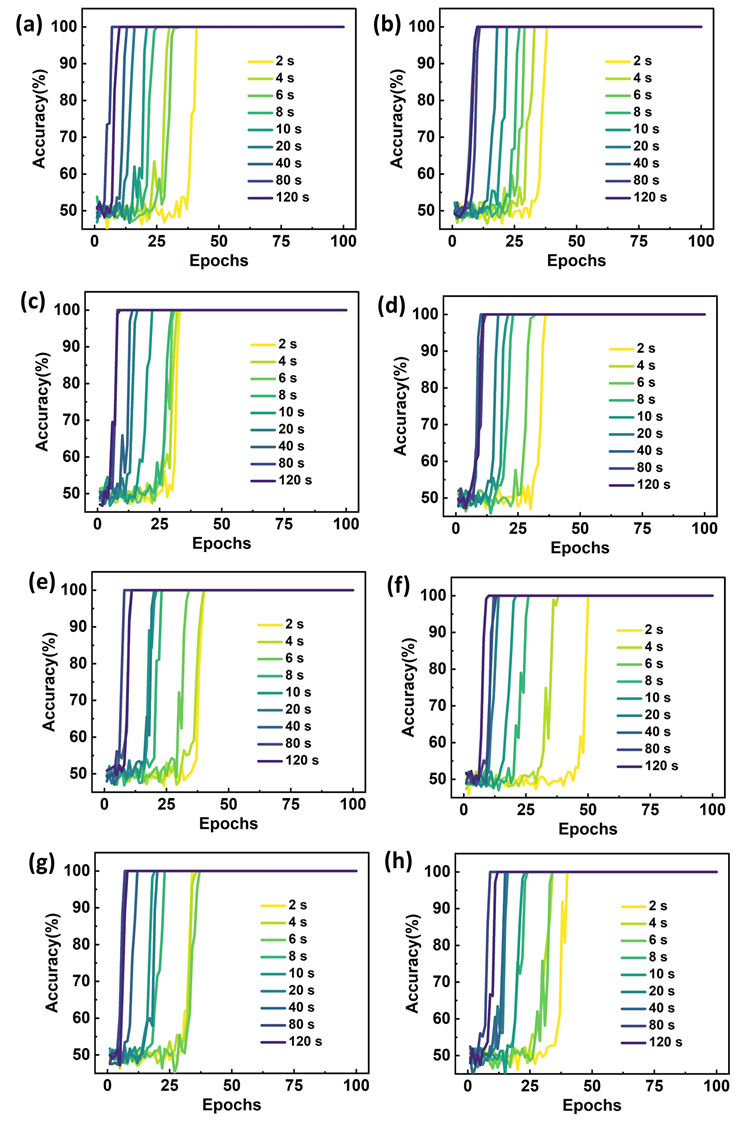
**

**Figure S13.** Comparisons of recognition rate at 8 different simulation running cycles.

8 random running cycles are compared as shown in Figure S13. It can be clearly found that the recognition rate is improved as the irradiation time increases for all of the running cycles, suggesting the repeatability of the simulation.


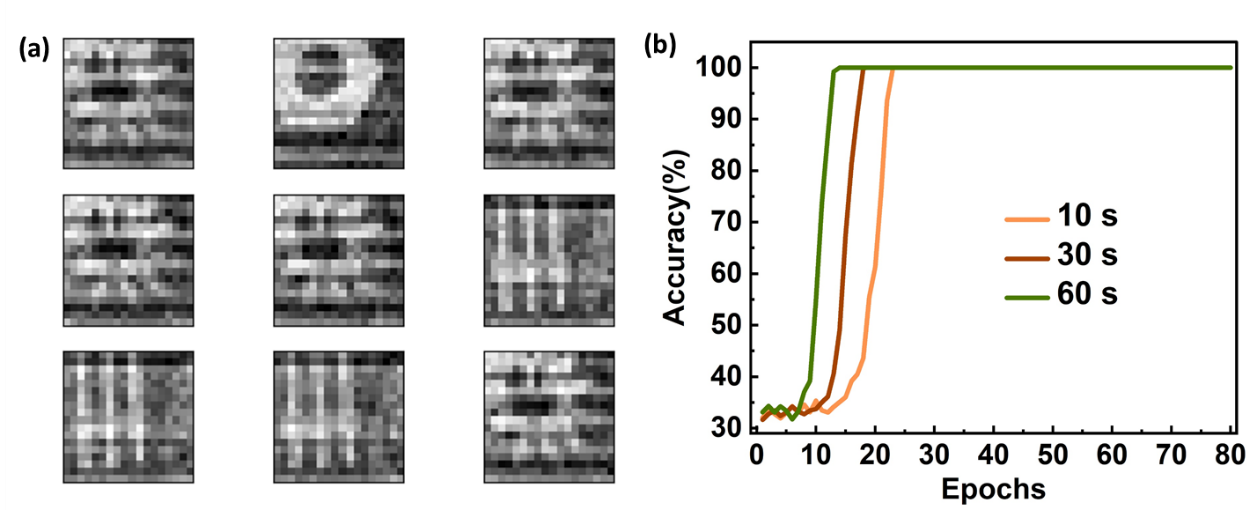


**Figure S14.** (a) Examples of the three objects including pin, wire and screw in a new image dataset. (b) Comparisons of the image recognition rate under different preprocessing time.

Similar method has been applied for the classification task using the three objects shown in Figure 6 in the main text. The image dataset is constructed according to the above three steps, where 1200 images are generated with the addition of random Gaussian noises. Representative images are shown in Figure S14a. The classification rate shows an apparent improvement as irradiation time increases (Figure S14b).

**References**

1. Campillo-Rivera, G. E.; Torres-Cortes, C. O.; Vazquez-Bañuelos, J.; Garcia-Reyna, M. G.; Marquez-Mata, C. A.; Vasquez-Arteaga, M.; Vega-Carrillo, H. R. X-Ray Spectra and Gamma Factors from 70 to 120 kV X-Ray Tube Voltages. *Radiation Physics and Chemistry* **2021**, *184*, 109437.
2. Blinov, N. N.; Vasil’ev, V. N.; Gromov, G. D.; Zhutyaev, S. G.; Lebedev, L. A.; Sidorin, V. P.; Stavitskii, R. V. Effect of the Anode Current of an X-Ray Tube on the Intensity and Energy Spectrum of Its Radiation. *Biomed. Eng.* **1984**, *18* (3), 96–99.
3. Jiang, J.; Xiong, M.; Fan, K.; Bao, C.; Xin, D.; Pan, Z.; Fei, L.; Huang, H.; Zhou, L.; Yao, K.; Zheng, X.; Shen, L.; Gao, F. Synergistic Strain Engineering of Perovskite Single Crystals for Highly Stable and Sensitive X-Ray Detectors with Low-Bias Imaging and Monitoring. *Nat. Photon.* **2022**, *16* (8), 575–581.
4. Zhuang, R.; Cai, S.; Mei, Z.; Liang, H.; Zhao, N.; Mu, H.; Yu, W.; Jiang, Y.; Yuan, J.; Lau, S.; Deng, S.; Han, M.; Jin, P.; Wang, C.; Zhang, G.; Lin, S. Solution-Grown BiI/BiI_3_ van Der Waals Heterostructures for Sensitive X-Ray Detection. *Nat. Commun.* **2023**, *14* (1), 1621.
5. Wei, W.; Zhang, Y.; Xu, Q.; Wei, H.; Fang, Y.; Wang, Q.; Deng, Y.; Li, T.; Gruverman, A.; Cao, L.; Huang, J. Monolithic Integration of Hybrid Perovskite Single Crystals with Heterogenous Substrate for Highly Sensitive X-Ray Imaging. *Nat. Photon.* **2017**, *11* (5), 315–321.
6. Han, M.; Xiao, Y.; Zhou, C.; Xiao, Z.; Tan, W.; Yao, G.; Wu, X.; Zhuang, R.; Deng, S.; Hu, Q.; Yang, Y.; Tang, Z.; Zhou, X.; Lin, H.; Liang, H.; Lin, S.; Mei, Z.; Wang, C.; Chen, Q.; Zhang, W.; Jiang, Y. Suppression of Ionic and Electronic Conductivity by Multilayer Heterojunctions Passivation Toward Sensitive and Stable Perovskite X‐Ray Detectors. *Adv. Funct. Mater.* **2023**, 2303376.
7. Temiño, I.; Basiricò, L.; Fratelli, I.; Tamayo, A.; Ciavatti, A.; Mas-Torrent, M.; Fraboni, B. Morphology and Mobility as Tools to Control and Unprecedentedly Enhance X-Ray Sensitivity in Organic Thin-Films. *Nat. Commun.* **2020**, *11* (1), 2136.
8. Kim, Y. C.; Kim, K. H.; Son, D.-Y.; Jeong, D.-N.; Seo, J.-Y.; Choi, Y. S.; Han, I. T.; Lee, S. Y.; Park, N.-G. Printable Organometallic Perovskite Enables Large-Area, Low-Dose X-Ray Imaging. *Nature* **2017**, *550* (7674), 87–91.
9. Deumel, S.; Van Breemen, A.; Gelinck, G.; Peeters, B.; Maas, J.; Verbeek, R.; Shanmugam, S.; Akkerman, H.; Meulenkamp, E.; Huerdler, J. E.; Acharya, M.; García-Batlle, M.; Almora, O.; Guerrero, A.; Garcia-Belmonte, G.; Heiss, W.; Schmidt, O.; Tedde, S. F. High-Sensitivity High-Resolution X-Ray Imaging with Soft-Sintered Metal Halide Perovskites. *Nat. Electron.* **2021**, *4* (9), 681–688.
10. Zhuang, R.; Wang, X.; Ma, W.; Wu, Y.; Chen, X.; Tang, L.; Zhu, H.; Liu, J.; Wu, L.; Zhou, W.; Liu, X.; Yang, Y. (Michael). Highly Sensitive X-Ray Detector Made of Layered Perovskite-like (NH_4_)_3_Bi_2_I_9_ Single Crystal with Anisotropic Response. *Nat. Photonics* **2019**, *13* (9), 602–608.
11. Choquette, M.; Rougeot, H.; Martin, J.-P.; Laperriere, L.; Shukri, Z.; Polischuk, B. T. Direct Selenium X-Ray Detector for Fluoroscopy, R&F, and Radiography; Dobbins III, J. T., Boone, J. M., Eds.; San Diego, CA, **2000**; p 128.
12. Chen, J.; Tang, H.; Liu, B.; Zhu, Z.; Gu, M.; Zhang, Z.; Xu, Q.; Xu, J.; Zhou, L.; Chen, L.; Ouyang, X. High-Performance X-Ray Detector Based on Single-Crystal β-Ga_2_O_3_:Mg. *ACS Appl. Mater. Interfaces* **2021**, *13* (2), 2879–2886.
